# Inheritance and Fitness Costs of Laboratory-Selected Resistance to Gpp34/Tpp35Ab1 Corn in Western Corn Rootworm (Coleoptera: Chrysomelidae)

**DOI:** 10.1093/jee/toad022

**Published:** 2023-02-17

**Authors:** Eliott M Smith, Ram B Shrestha, Aaron J Gassmann

**Affiliations:** Department of Plant Pathology, Entomology and Microbiology, Iowa State University, Ames, IA 50011, USA; Department of Plant Pathology, Entomology and Microbiology, Iowa State University, Ames, IA 50011, USA; Department of Plant Pathology, Entomology and Microbiology, Iowa State University, Ames, IA 50011, USA

**Keywords:** Bt crop, corn, insect resistance management, population genetics, refuge strategy

## Abstract

Western corn rootworm, *Diabrotica virgifera virgifera* LeConte (Coleoptera: Chrysomelidae), is a serious pest of corn and is currently managed with corn hybrids that produce insecticidal proteins derived from the bacterium *Bacillus thuringiensis* (Bt). Bt corn kills rootworm larvae and reduces larval feeding injury to corn roots. The Bt protein Gpp34/Tpp35Ab1, previously named Cry34/35Ab1, has been widely used in transgenic Bt corn for management of western corn rootworm, and field-evolved resistance has been found in some populations. In the United States, the refuge strategy is used to manage Bt resistance, with refuges of non-Bt host plants serving as a source of Bt-susceptible individuals, which in turn reduce the frequency of homozygous resistant individuals within a population. As such, the dominance of resistance strongly influences resistance evolution, with faster evolution of resistance when resistance is not recessive. Additionally, selection for resistance by a Bt crop leads to the accumulation of resistance alleles within refuge populations, thereby reducing the capacity of refuges to delay resistance. However, fitness costs can remove resistance alleles from refuge populations and preserve the dynamic of refuges producing Bt-susceptible genotypes. Bt-susceptible and Gpp34/Tpp35Ab1-resistant western corn rootworm were used to quantify the inheritance and fitness costs of resistance. We found that Gpp34/Tpp35Ab1 resistance was not recessive and had the accompanying fitness costs of slower developmental rate to adulthood and lower egg viability. This research will help improve insect resistance management by providing a better understanding of the risk of western corn rootworm evolving resistance to transgenic corn that produces Gpp34/Tpp35Ab1.

The western corn rootworm, *Diabrotica virgifera virgifera* LeConte (Coleoptera: Chrysomelidae), is a serious pest of corn *Zea mays* L. and is widely distributed throughout the midwestern United States (US) and parts of Europe (Toepfer and Kuhlmann 2006, Meinke et al. 2009, Dillen et al. 2010, Gassmann 2021). This univoltine pest causes over two billion dollars of economic losses annually to US farmers, and this arises because of both yield losses and costs associated with management (Wechsler and Smith 2018). The primary cause of yield loss is from larval feeding on corn roots, which interferes with a plant’s ability to acquire water and nutrients, and makes plants more susceptible to lodging (Gray and Steffey 1998, Oleson et al. 2005). Additionally, adults can hinder fertilization and seed set when they feed on corn ears and silk, and this feeding can reduce yield in some cases (Meinke et al. 2021). Farmers have utilized multiple tactics to manage this pest, including crop rotation, foliar insecticides, soil-applied insecticides, and plant incorporated protectants (Meinke et al. 1998, Levine et al. 2002, Gray et al. 2009, Tinsley et al. 2015, Gassmann 2021). Since larval feeding injury causes the greatest yield loss, most management practices focus on suppressing larval abundance and associated feeding injury (Vidal et al. 2005, Meinke et al. 2021).

In 1901, and independently in 1911, the bacteria *Bacillus thuringiensis* Berliner (Bt) was found to induce disease symptoms in specific insects (Sanahuja et al. 2011). Since 1938, this bacterium has been used in crop protection, with current management practices using Bt proteins as plant incorporated protectants in genetically engineered crops (Sanahuja et al. 2011). There have been four commercially released Bt traits for the management of western corn rootworm: Cry3Bb1, mCry3A, eCry3.1Ab, and Cry34/35Ab1 (US EPA 2010a, b, c, 2012; Kelker et al. 2014; Zukoff et al. 2016). The first commercially available Bt traits for the management of western corn rootworm was Cry3Bb1, which became available in 2003. Cry34/35Ab1, which has been renamed to Gpp34/Tpp35Ab1 (Crickmore et al. 2021), was registered by the United States Environmental Protection Agency (US EPA) in 2005, followed by mCry3A and eCry3.1Ab in 2006 and 2012, respectively (Gassmann 2016).

Field-evolved resistance by the western corn rootworm has been documented for all four of the Bt traits currently available for rootworm management (Gassmann 2021). In 2009, the first instances of field-evolved resistance to Cry3Bb1 corn were documented in Iowa (Gassmann et al. 2011). Later, cross-resistance was documented among Cry3Bb1, mCry3A, and eCry3.1Ab (Gassmann et al. 2014, Jakka et al. 2016). By 2013, fields in Iowa and Minnesota planted to Bt corn treated with Gpp34/Tpp35Ab1 exhibited high levels of feeding injury from western corn rootworm. Subsequent bioassays revealed that these populations had evolved resistance to Gpp34/Tpp35Ab1 corn, although resistance was incomplete, with survival on Bt corn less than on non-Bt corn (Gassmann et al. 2016, Ludwick et al. 2017). By 2017, a western corn rootworm population was identified with similar survival on Gpp34/Tpp35Ab1 corn and non-Bt corn, suggesting complete resistance (Gassmann et al. 2020).

The refuge strategy has been used globally, including in Australia, India, South Africa, and the United States to delay the evolution of pest resistance to Bt crops (Gould 1998, Tabashnik et al. 2013, US EPA 2021). A Bt refuge is any vegetation that supports larval development but does not impose selection for Bt resistance (Gould 1998). Therefore, a refuge may be naturally occurring host plants in the landscape, a portion of a field planted to non-Bt plants, or non-Bt plants interspersed among Bt plants within a field (Gould 1998, Deitloff et al. 2016). In corn-growing regions of the United States, the US EPA has set the refuge requirement for corn as 5–20% non-Bt plants depending on the product and refuge type (US EPA 2021). Under the refuge strategy, resistance evolution is delayed because susceptible individuals from the refuge plants disperse and mate with resistant individuals developing on Bt plants, resulting in heterozygous offspring. Resistance is delayed, compared to a scenario when refuges are absent, if these heterozygous progeny have lower fitness on a Bt crop than homozygous resistant individuals (Tabashnik et al. 2008, Gassmann 2021).

The success of the refuge strategy can be affected by the dominance of resistance and fitness costs of resistance (Tabashnik et al. 2008, Gassmann et al. 2009, Carrière et al. 2012). Dominance of Bt resistance is a measure of survival for heterozygotes exposed to Bt plants compared to that of homozygous resistant individuals (Bourguet et al. 2000). Fitness costs can be measured as the difference in a fitness parameter (e.g., survival) between resistant and susceptible genotypes in the absence of Bt (Gassmann et al. 2009). In general, there is a positive relationship between the dominance of resistance and the rate of resistance evolution, with resistance evolving more quickly for nonrecessive resistance compared with cases when resistance is recessive (Tabashnik et al. 2004). In contrast, fitness costs act to delay the evolution of resistance by imposing selection against resistant individuals in the absence of Bt (i.e., in refuges) and preserving the dynamic of refuges producing Bt-susceptible individuals (Gassmann et al. 2009). Previous research on Cry3Bb1 resistance in western corn rootworm indicated that resistance is often nonrecessive with minimal fitness costs (Petzold-Maxwell et al. 2012, Ingber and Gassmann 2015, Paolino and Gassmann 2017). Consequently, nonrecessive resistance and a lack of fitness costs may have contributed to field-evolved resistance to Cry3Bb1 corn in Iowa and other parts of the Midwest (Gassmann 2021, Gassmann and Reisig 2023).

In this study, we quantified dominance of resistance and tested for the presence of fitness costs using a western corn rootworm strain with laboratory-selected resistance to Gpp34/Tpp35Ab1 corn. Based on past research on resistance to Cry3Bb1 corn by western corn rootworm, we hypothesized that resistance would be nonrecessive and that minimal fitness costs would be present. Paddock et al. (2021) also studied fitness costs for laboratory-selected resistance to Gpp34/Tpp35Ab1 in western corn rootworm, but the current study represents an advance in knowledge because it uses genetically similar resistant and susceptible strains (Gassmann et al. 2009). Additionally, this work reports the dominance of resistance to Gpp34/Tpp35Ab1 corn by western corn rootworm, for which very little information is currently available. Data from this study will aid in refining strategies to manage resistance to Gpp34/Tpp35Ab1 by the western corn rootworm and aid in assessing the risk of resistance evolving to Gpp34/Tpp35Ab1 in additional populations.

## Materials and Methods

### Insect Strains

The parental strain of western corn rootworm used in this research was generated by collecting adult rootworm from fields in Dodge City, KS, Caledonia, MN, and Janesville, WI, between 2002 and 2004. These field-collected strains were crossed with a nondiapausing laboratory strain at USDA-ARS Plant Genetic Research Laboratory in Colombia, MO, and subsequently pooled in 2009 to generate a parental strain (Meihls et al. 2008, Deitloff et al. 2016). In 2010, this parental strain was sent to Iowa State University where it was divided into two strains: 1) Bt-susceptible strain and 2) Bt-resistant strain.

The Bt-susceptible strain was maintained on non-Bt corn and was never exposed to Bt corn. The Bt-resistant strain was selected for resistance on Gpp34/Tpp35Ab1 corn by rearing larvae from neonate to pupation on seedling mats of Gpp34/Tpp35Ab1 corn (event 59122-7; Corteva Agriscience, Indianapolis, IN) for 26 generations between the F1 and F47, and then rearing continuously on Gpp34/Tpp35Ab1 corn for eight generations (F48 to F55). Additionally, the Bt-resistant strain was back-crossed with the Bt-susceptible strain four times (F27, F36, F43, and F44) to increase the genetic similarity between the resistant and susceptible strains. Before initiating experiments, the Bt-resistant strain was reared on non-Bt corn for three generations (F56, F57, and F58) to avoid any sub-lethal maternal or paternal effects of Bt corn. Both strains were maintained at an average population size of between 2,000 and 2,500 adults per generation, although the population size of the Bt-resistant strain tended to be somewhat smaller than this average population size during generations of selection (~1,750 adults per generation). Population sizes were smaller during generations of selection because Bt corn killed larval insects.

### Rearing of Strains

Adult insects were held in mesh cages (30 × 30 × 30 cm, MegaView Science Co. Ltd., Taichung, Taiwan) housed in a biological incubator (I41-LL Percival Scientific, Perry, IA; 25°C; 16:8 [L:D] hr). Adults were provided with a complete adult diet (Bio-Serv, Frenchtown, NJ), 1.5% agar solid as a source of water, and corn leaf tissue. Food and water resources were replaced three times per week. Eggs were collected in an oviposition dish, which was prepared by covering the bottom of a 10 cm Petri dish with a 5 mm layer of sieved field soil (<80 mesh particle size). A piece of aluminum foil was then loosely placed over the Petri dish to generate a darkened environment to stimulate oviposition. After 6–10 d, eggs were washed from the soil in a 60-mesh sieve. Eggs were then added to seedling mats following Ingber and Gassmann (2015). Briefly, seedling mats contained 25 ml corn seeds, 80 ml water, and 500 ml of a 1:1 mixture of field soil and potting medium (Sunshine LC1 Professional Growing Mix, SunGro Horticulture, Vancouver, BC, Canada), and were held in a 1L clear plastic container with a lid (Dart C32DE, Dart Container Corporation, Mason, MI). Eggs were added to seedling mats at a density of 900 eggs per seedling mat during generations of selection with Bt corn, and a density of 600 eggs per seedling mat during generations on non-Bt corn. These smaller seedling mats were inverted, removed from their containers, and placed on top of larger seedling mats, which were held in 6 liter plastic boxes with lids (Sterlite 1642, Sterlite Corporation, Townsend, MA). Larger seedling mats contained 120 ml corn seeds, 500 ml water, and 3 liters of the soil mixture; and larvae completed development in these larger seedling mats. The larger seedling mats were at least 7 d old before the smaller seedling mats were transferred onto them, and larger seedling mats contained the same type of corn seed (i.e., non-Bt corn or Gpp34/Tpp35Ab1 corn) used in smaller seedling mats. A fabric cover under the lid was used to prevent larvae and adults from escaping. Upon emergence, adults were collected using an aspirator attached to a vacuum pump (1531-107B-6557x, Gast Manufacturing Inc., Benton Harbor, MI.) and adults were then placed inside a mesh cage.

### Dominance of Resistance

This experiment was conducted between December 2019 and April 2020 and used the F58 of the Bt-resistant strain and the F60 of the Bt-susceptible strain. Two heterozygous crosses were made by reciprocally crossing unmated adults from the Bt-resistant and Bt-susceptible strains (i.e., resistant ♂ × susceptible ♀; susceptible ♂ × resistant ♀). Adults used for crosses were collected from non-Bt seedling mats within four hours of emergence to ensure they were unmated, following Shrestha and Gassmann (2020). Adults were held individually in small Petri dishes until sex could be determined by examining the basitarsi morphology following Hammack and French (2007) after which, adults were added in a 1-to-1 ratio to a small mesh cage (17.5 × 17.5 × 17.5 cm; 4S1515, BugDorm Store, Taiwan). The resistant ♂ × susceptible ♀ dorm received 710 total adults; the susceptible ♂ × resistant ♀ dorm received 822 adults. Simultaneously with the collection of eggs from the heterozygous crosses, eggs were collected from the Bt-resistant and Bt-susceptible strains. Eggs from each cross and the parental strains were held in a biological incubator (26°C, 40–60% RH, 16:8 [L:D] hr) until larval eclosion.

Each of the four genotypes (Bt-resistant, Bt-susceptible, resistant ♂ × susceptible ♀, and susceptible ♂ × resistant ♀) were added singly to seedling mats of two corn hybrids: Gpp34/Tpp35Ab1 corn (P8954XR) or the non-Bt isoline to Gpp34/Tpp35Ab1 corn (P8954R), with the presence and absence of Gpp34/Tpp35Ab1 confirmed, at the time each bag of seed was opened, with an ELISA based on a kit (Envirologix, Portland, ME). Sixteen replications were used for each combination of rootworm genotype by corn hybrid for a total of 128 seedling mats. Methods followed Ingber and Gassmann (2015) and used a two-stage, seedling-mat rearing method, similar to the methods applied for the rearing of strains, except that the seedling mats were smaller. Briefly, 18 neonates (<24 hr old) were placed on the exposed roots of a 5 d old small seedling mat (15 ml corn seeds, 50 ml water, and 200 ml soil mixture) placed in a cylindrical 500 ml container (DeliPRO TD40016, TRiPAK Industrial USA, LLC, White Plains, NY). Small seedling mats with larvae were then placed in a biological incubator (25°C; 60% RH; 16:8 [L:D] hr) for 10 d. After 10 d, each small seedling mat was placed on top of a 5 d old large seedling mat (25 ml corn seed, 80 ml water, and 500 ml soil mixture) placed in a 1 liter rectangular container (C32DE container, Dart Container Corp., Mason, MI), which was covered with white chiffon fabric and a lid with holes added for ventilation. These large seedling mats were checked every 2 to 3 d for adult emergence, with the number of adults emerging per day per seedling mat recorded.

### Fitness Costs of Resistance

This experiment was conducted between February and July 2020 and used the F58 of the Bt-resistant strain and F61 of the Bt-susceptible strain. Fitness costs were measured for six life-history traits: 1) survival from the larval to the adult stage, 2) developmental rate, 3) longevity, 4) adult head capsule width, 5) fecundity, and 6) egg viability. A total of 20 replicates were established for both the Bt-resistant and Bt-susceptible strains. A replicate consisted of a single non-Bt seedling mat prepared following the methods described in Ingber and Gassmann (2015). A total of 18 neonate larvae were placed on each seedling mat, and adults were collected three times per week. Proportion survival to adulthood was calculated as the number of adults collected per seedling mat divided by the number of larvae added. Upon emergence, live adults were placed individually in Petri dishes and examined under a stereo-microscope (MZ6, Leica Microsystems, Wetzlar, Germany), with sex determined following Hammack and French (2007). Developmental rate was calculated per replicate as the average number of days between when larvae were added to seedling mats and the emergence of adults for each sex.

All adults that emerged from a single seedling mat were added to the same small mesh cage (17.5 × 17.5 × 17.5 cm; 4S1515, BugDorm Store, Taiwan), and cages were held in a biological incubator (25°C; 60% RH; 16:8 [L:D] hr). Adults were supplied with a complete diet, 1.5% agar solid, and corn leaf tissue, all of which were replaced every 3 to 4 d. When fresh food was provided, any dead adults were removed and placed in 85% ethanol. Adult longevity was determined for all adults and was calculated by subtracting the average day of adult emergence by sex within a replicate from the day on which each individual died. After death, head capsule width was measured as the outermost boundary between the compound eyes under a stereo-microscope using a digital camera (Motic North America, BC, Canada) with image analysis software (Motic Images Advanced V3.0 software, Motic North America, BC, Canada). For each specimen, sex was also determined following Hammack and French (2007).

Each cage received a 100 mm oviposition dish, which was replaced every 7 d. Each oviposition dish was washed following the procedures used for strain rearing. Fecundity per female per replicate was calculated as total eggs per cage divided by the total number of females in that cage. All but 25 eggs from each week’s oviposition dish were stored in 70% ethanol. Stored eggs were quantified by photographing eggs, which were evenly distributed in a Petri dish, under a stereo-microscope and manually counting the photographed eggs within the ImageJ software (Schneider et al. 2012).

The 25 eggs that were set aside were used to determine egg viability. For each of the 40 small cages with adults, weekly cohorts of 25 eggs were assessed for egg viability. To quantify viability, eggs were placed on a 1.5% agar solid in a 50 mm Petri dish and examined three times per week under a stereo-microscope for newly eclosed larvae. All larvae were removed once counted. Petri dishes were checked for larvae until no new larvae eclosed for four consecutive observation periods. Egg viability was calculated as the number of larvae that eclosed divided by the number of eggs evaluated. During this process, eggs were held in a biological incubator (25°C, 60% RH, 0:24 [L:D] hr). On average, egg viability was measured for 3.7 ± 0.8 (mean ± standard deviation) cohorts of eggs for each replicate. For one replicate with the Bt-resistant strain, we were unsuccessful in obtaining a measurement of egg viability because this replicated did not produce enough eggs to measure viability.

### Data Analysis

All data were analyzed with SAS 9.4 (SAS Institute 2015). Data on proportion survival to adulthood from the experiment measuring the dominance of resistance were analyzed with an analysis of variance (ANOVA) based on a general linear model (PROC GLM) (Sokal and Rohlf 1995). Proportion survival was calculated for each replicate (i.e., seedling mat) as the number of adults that emerged from a seedling mat divided by the number of neonate larvae added to that seedling mat. Rootworm genotype, corn hybrid, and the interaction between genotype and hybrid were the fixed effects in the model. Data on proportion survival were first analyzed to test for a difference between the heterozygous crosses using an ANOVA model that included the factors of corn hybrid, heterozygous crosses, and their interaction. There was no significant interaction between the heterozygous crosses and corn hybrid (*F*_1,60_ = 0.02, *P* = 0.88) or significant effect of heterozygous crosses (*F*_1,60_ = 0.77, *P* = 0.38), and consequently, the heterozygous crosses were pooled in subsequent analyses. Subsequently, the full data set was analyzed with an ANOVA that included the factors of genotype (Bt-resistant, Bt-susceptible, and heterozygotes), corn hybrid, and their interaction. A significant genotype-by-hybrid interaction was present, so comparisons were made among genotypes within each hybrid and between hybrids for each genotype, with a significance level of *P* < 0.0056 after a Bonferroni adjustment using nine comparisons (Sokal and Rohlf 1995). Corrected survival was calculated, based on the formula of Abbott (1925), as the quotient of proportion survival on Bt corn divided by the proportion survival on non-Bt corn. The dominance of resistance (*h*) was calculated following Liu and Tabashnik (1997) using the corrected survival from each genotype (*h* = [heterozygote – Bt-susceptible] ÷ [Bt-resistant – Bt-susceptible]), with 0 = recessive, 1 = dominant, and 0.5 = additive inheritance.

For the fitness cost experiment, data were analyzed with a mixed-model ANOVA (PROC MIXED). For developmental rate, adult head capsule width and adult longevity, the ANOVA model included the fixed effects of strain, sex, and their interaction, and random effects were replicate (i.e., cage or seedling mat) nested within strain and sex crossed with replicate nested within strain. For survival to adulthood, egg viability, and fecundity, the ANOVA model included the fixed effects of strain, sex, and their interaction, and random effect of replicate (i.e., cage or seedling mat) nested within strain. When significant differences were present between strains, the magnitude of a fitness cost was calculated as the percentage difference between strains using the following formula of Gassmann et al. (2009): [|(fitness of susceptible – fitness of resistant)| ÷ fitness of susceptible] × 100.

## Results

**Fig. 1.**
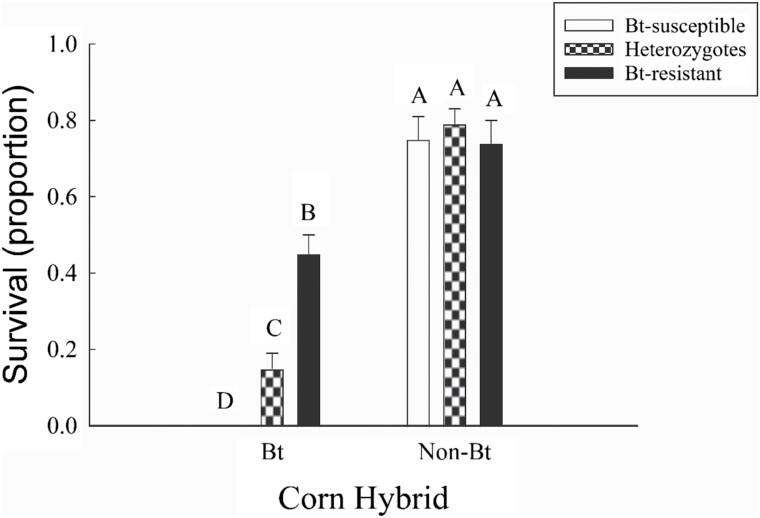
Proportion survival to adulthood of western corn rootworm on Gpp34/Tpp35Ab1 corn and the non-Bt near isoline for the Bt-resistant, Bt-susceptible, and heterozygous genotypes (*n* = 16 seedling mat bioassays per treatment). Bar heights represent sample means, and error bars are the standard error of the mean. Capital letters denote significant differences between means.

Survival to adulthood in the inheritance experiment was affected by a significant interaction between genotype and hybrid (Fig. 1; Table 1). All three genotypes (i.e., resistant, susceptible, and heterozygotes) had similar survival on non-Bt corn. By contrast, on Gpp34/Tpp35Ab1 corn, survival was highest for resistant individuals, intermediate for heterozygotes, and lowest for the Bt-susceptible strain. Additionally, survival of all three strains (Bt-resistant, heterozygotes, and Bt-susceptible) was significantly lower on Bt corn compared to non-Bt corn. Corrected proportion survival on Gpp34/Tpp35Ab1 seedling mats was 0.67 for the Bt-resistant strain, 0.24 for heterozygotes, and 0 for the Bt-susceptible strain. Based on these values, the dominance of resistance was 0.36 ([0.24 – 0] ÷ [0.67 – 0]).

**Table 1.** Analysis of variance for survival to adulthood

Effect^*a*^^,^^*b*^	df	*F*-Statistic	*P*
Genotype	2, 122	12.70	<0.0001
Hybrid	1, 122	287.96	<0.0001
Genotype × Hybrid	2, 122	15.90	<0.0001

^*a*^Genotypes tested were Bt-resistant, Bt-susceptible, and heterozygotes.

^*b*^Corn hybrids tested were non-Bt corn and Gpp34/Tpp35Ab1 corn.

**Fig. 2.**
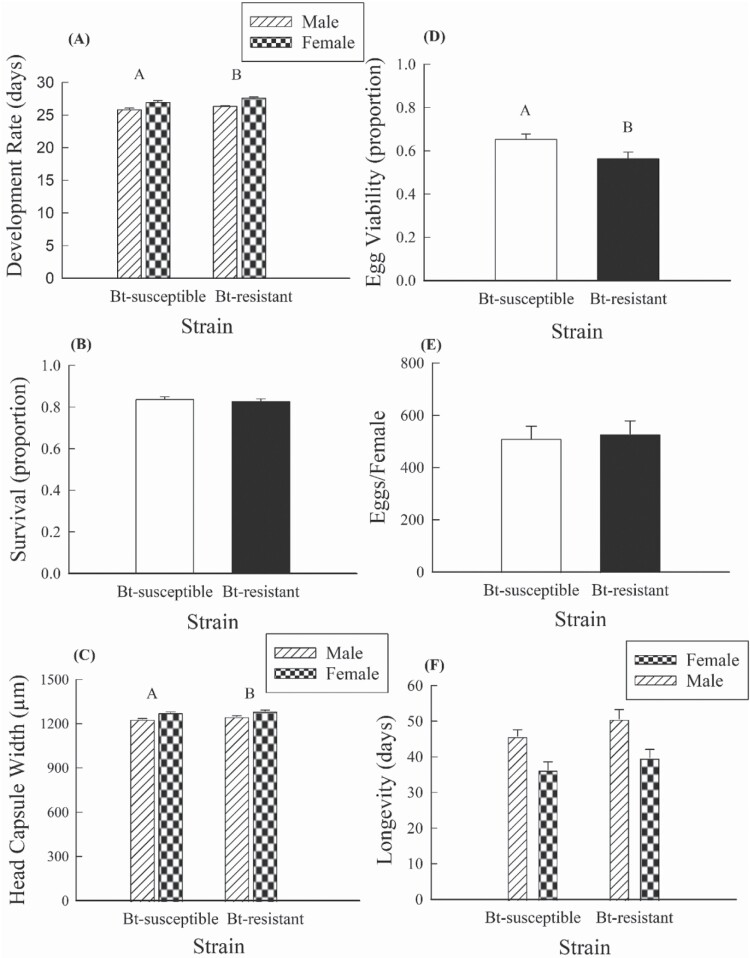
Comparison of life-history data for the Bt-resistant and Bt-susceptible western corn rootworm strains reared on seedling mats of non-Bt corn (*n* = 20 seedling mats per strain). Bar heights represent sample means, and error bars are the standard error of the mean. Data are presented for (A) developmental rate, (B) proportion survival to adulthood, (C) adult head capsule width, (D) egg viability, (E) average eggs per female, and (F) adult longevity. Capital letters denote significant differences between strains.

For the fitness cost experiment, which was conducted using only non-Bt seedlings mats, Bt-resistant insects took significantly longer to develop from neonate to adult than Bt-susceptible individuals (Bt-resistant = 27.0 ± 0.0956; Bt-susceptible = 26.3 ± 0.231; mean ± standard error of the mean), which represents a fitness cost of 2.66% ([|(26.3 – 27.0)| ÷ 26.3] × 100) (Fig. 2A; Table 2). Additionally, females took significantly longer to develop to adulthood than males (Fig. 2A; Table 2). Proportion egg viability was significantly lower for the Bt-resistant insects (0.559 ± 0.031) compared to Bt-susceptible insects (0.662 ± 0.030), which represents a fitness cost of 15.6% ([|(0.662 – 0.559)| ÷ 0.662] × 100) (Fig. 2D; Table 2). Adult size, measured as head capsule width, showed a significant effect for both strain and sex (Table 2). Resistant insects (1270 ± 3.14 µm) were significantly larger than Bt-susceptible insects (1250 ± 2.79), which represents a fitness benefit of 1.60% ([|(1250 – 1270)| ÷ 1250] × 100), and females were significantly larger than males (Fig. 2C). For adult longevity, males lived significantly longer than females, although no effect of strain was detected (Fig. 2F; Table 2). No other significant effects were detected for any of the life-history traits measured.

**Table 2.** Analysis of variance for life-history traits of the Bt-resistant and the Bt-susceptible genotypes reared on non-Bt corn

Life-History Trait	Effect	df	*F*-Statistic	*P*
Developmental rate	Strain	1, 38	4.35	0.0438
	Sex	1, 38	140.17	<0.0001
	Strain × Sex	1, 38	0.29	0.5946
Proportion Survival	Strain	1, 38	1.18	0.2852
Head capsule width	Strain	1, 38	11.69	0.0015
	Sex	1, 38	147.20	<0.0001
	Strain × Sex	1, 38	1.42	0.2414
Egg Viability	Strain	1, 37	5.72	0.0220
Fecundity	Strain	1, 38	0.07	0.7939
Adult Life Span	Strain	1, 38	2.27	0.1401
	Sex	1, 38	27.01	<0.0001
	Strain × Sex	1, 38	0.09	0.7663

## Discussion

We found that the Bt-resistant strain exhibited nonrecessive inheritance of resistance and fitness costs of reduced egg viability and a longer developmental rate. Mathematical modeling and previous empirical research with western corn rootworm support the idea that more dominant resistance contributed to a more rapid evolution of resistance (Onstad and Meinke 2010). The dominance value obtained in this study (*h* = 0.36) indicates nonrecessive inheritance of resistance, with resistant heterozygotes capable of surviving on Gpp34/Tpp35Ab1 corn (Fig. 1), therefore there is a heightened risk of resistance evolution in the field (Roush 1997, Onstad and Meinke 2010, Tabashnik and Gould 2012, Tabashnik and Carrière 2014, Andow et al. 2016). Nonrecessive inheritance of resistance to Cry3Bb1 corn by western corn rootworm was found in several studies (Meihls et al. 2008, Petzold-Maxwell et al. 2012, Ingber and Gassmann 2015, Paolino and Gassmann 2017), and these data suggest that this pattern may also extend to Gpp34/Tpp35Ab1 corn. It is noteworthy that field-evolved resistance to Gpp34/Tpp35Ab1 corn has been identified in several populations of western corn rootworm within eight years after this Bt trait was released (Gassmann et al. 2016, 2020; Ludwick et al. 2017; Reinders and Meinke 2022).

Nonrecessive inheritance of Bt resistance results from, and is an indication of, a Bt crop producing less than a high dose of Bt toxin (Storer et al. 2006, Huang et al. 2011). A high dose Bt crop is defined as one that produces 25 times more Bt toxin than is needed to kill susceptible larvae, a toxin concentration that kills 99.99% of susceptible larvae, or a concentration that kills 95% of heterozygotes (US EPA 1998, Tabashnik et al. 2008). Resistance is expected to evolve more quickly when insects are not exposed to a high dose of a toxin (Tabashnik et al. 2008, 2013). Gpp34/Tpp35Ab1 corn does not achieve a high dose against the western corn rootworm (Storer et al. 2006, Petzold-Maxwell et al. 2013, Shrestha et al. 2016). Other instances of nonrecessive resistance to Bt toxins in transgenic crops include corn earworm *Helicoverpa zea* Boddie (Lepidoptera: Noctuidae), fall armyworm *Spodoptera frugiperda* (J. E. Smith) (Lepidoptera: Noctuidae), and maize stalk borer *Busseola fusca* (Fuller) (Lepidoptera: Noctuidae) (Campagne et al. 2013, Yang et al. 2020, Huang 2021, Santiago‐González et al. 2022). In these previous examples, the Bt trait was not considered high dose. In contrast to these examples, European corn borer *Ostrinia nubilalis* (Hübner) (Lepidoptera: Crambidae) has remained susceptible to Cry1Ab and Cry1F, in most instances, including in the United States and Europe, because resistance generally evolves slower when the high-dose criterion is met; however, resistance to Cry1F corn has been found for populations of this pest in Canada (Huang et al. 2011, Siegfried and Hellmich 2012, Smith et al, 2019, García et al. 2023).

The results reported here show fitness costs associated with resistance to Gpp34/Tpp35Ab1, as manifested by delayed developmental rate from neonate to adult and reduced egg viability (Table 2; Fig 2A and D). However, resistant individuals were significantly larger than susceptible individuals, an effect that might somewhat counterbalance effects of costs on developmental rate and egg viability (Table 2; Fig. 2C). While there is a direct relationship between reduced egg viability and fitness, the effects of developmental rate and size on fitness are less apparent. In the field, longer developmental rate may lead to higher rates of mortality from natural enemies (Price et al. 1980). By contrast, larger size for western corn rootworm males may increase male mating success (Murphy and Krupke 2011). As such, while reduced egg viability is expected to reduce fitness across environments, there is likely to be some ecological contingency as far as the extent to which developmental rate and adult size affect fitness, with factors including the prevalence of natural enemies and mate competition influencing effects on fitness.

Studies observing individual life-history parameters for Cry3Bb1-resistant strains of western corn rootworm failed to detect fitness costs for some strains (Petzold-Maxwell et al. 2012, Hoffmann et al. 2014, Ingber and Gassmann 2015, Paolino and Gassmann 2017) but found evidence of fitness costs in other cases (Hoffmann et al. 2015, Ingber and Gassmann 2015, Paolino and Gassmann 2017). Based on a selection experiment, St. Clair et al. (2020) found evidence of fitness costs of Cry3Bb1 resistance, as manifested by a decrease in resistance over multiple generations in the absence of Cry3Bb1. Paddock et al. (2021) reported evidence for fitness costs of Gpp34/Tpp35Ab1 resistance in western corn rootworm using a strain with laboratory-selected resistance. In general, fitness costs often accompany Bt resistance (Gassmann 2023). Gassmann et al. (2009) reported that 62% of selection experiments and 34% of experiments comparing life-history traits found evidence of fitness costs accompanying Bt resistance. Our research indicates the presence of fitness costs to Gpp34/Tpp35Ab1 resistance in a laboratory-selected rootworm strain, and to the extent that fitness costs also are associated with field-evolved resistance, they should act to delay the rate of resistance evolution.

Because this study used a strain with laboratory-selected resistance, a natural question that arises is how likely it is that the results reported here may extrapolate to the field. Groeters and Tabashnik (2000) reported that the genetic architecture (i.e., the number of loci conferring phenotypic resistance) was often similar between laboratory-selected resistance and field-evolved resistance, suggesting that the study of laboratory-selected resistance can be relevant to understanding resistance in the field. Additional insights into this question may be gained by comparing cases of field-evolved versus laboratory-selected resistance to Cry3Bb1 corn. Studies of two strains with laboratory-selected resistance found nonrecessive resistance while three out of four strains with field-evolved resistance also displayed nonrecessive resistance (Meihls et al. 2008, Petzold-Maxwell et al. 2012, Ingber and Gassmann 2015, Paolino and Gassmann 2017). Additionally, some studies of both laboratory-selected resistance and field-evolved resistance found evidence of fitness costs, while others did not (Petzold-Maxwell et al. 2012; Hoffmann et al. 2014, 2015; Ingber and Gassmann 2015; Paolino and Gassmann 2017). As such, the results obtained from this study appear likely to reflect at least some cases of field-evolved Gpp34/Tpp35Ab1 resistance. However, studying multiple strains with field-evolved resistance is needed to understand the inheritance of Gpp34/Tpp35Ab1 resistance more clearly, and the extent to which fitness costs may accompany such resistance.

The current study focused on fitness costs affecting homozygous susceptible individuals. However, the fitness of individuals that are heterozygous for resistance strongly influences the rate of resistance evolution because, when resistance first evolves, most resistance alleles occur in a heterozygous state (Roush 1997, Carrière and Tabashnik 2001, Gassmann 2023). Past research on western corn rootworm tested for fitness costs affecting heterozygous resistant individuals (i.e., nonrecessive fitness costs) using a strain with laboratory-selected resistance to Cry3Bb1 corn, and found evidence for a nonrecessive cost affecting adult size (Hoffmann et al. 2015). Additional research testing for the presence of nonrecessive fitness costs for western corn rootworm strains with field-evolved resistance to Gpp34/Tpp35Ab1 should help in further assessing risks associated with western corn rootworm evolving resistance to Gpp34/Tpp35Ab1.

Cases of successful resistance-management typically satisfy the high dose/refuge requirement (Huang et al. 2011, Matten et al. 2012, Tabashnik et al. 2013, Gassmann and Reisig 2023). Resistance can decline in populations, and such decline can be favored by fitness costs or negative cross-resistance. (Tabashnik 1994; Carrière and Tabashnik 2001; Tabashnik et al. 2009, 2013; Gassmann 2023). Although the fitness costs found in this study may act to delay the evolution of resistance, this effect could be counterbalanced by nonrecessive inheritance of resistance (Gassmann et al. 2009).

For Bt traits that do not achieve a high dose, pyramiding of multiple Bt traits can also be used to delay resistance, with insects that possess resistance to one trait killed by the second Bt trait in a pyramid and vice versa (Roush 1998; Carrière et al. 2015, 2016). Current Bt pyramids for management of corn rootworm include the combination of Gpp34/Tpp35Ab1 with either mCry3A or Cry3Bb1 (Gassmann 2021). However, these Bt pyramids were used in management after resistance to Cry3Bb1 and mCry3A had already evolved in the field, and this subsequently diminished the resistance management benefit of pyramiding (Gassmann and Reisig 2023). Field populations with resistance to Gpp34/Tpp35Ab1 typically display resistance to Cry3 toxins, which highlights how using Bt traits that are less than high dose singly, before pyramiding, can compromise the resistance management benefit of pyramiding (Gassmann et al. 2016, 2020; Ludwick et al. 2017; Reinders et al. 2022).

Resistance by western corn rootworm to Bt corn is broad and widespread, with reports of resistance to Cry3Bb1, mCry3A, eCry3.1Ab, and Gpp34/Tpp35Ab1 throughout the midwestern corn-growing region (Wangila et al. 2015; Gassmann 2016, 2021, 2021; Jakka et al. 2016; Zukoff et al. 2016; Ludwick et al. 2017; Calles-Torrez et al. 2019; Reinders and Meinke 2022; Reinders et al. 2022; Gassmann and Reisig 2023). The case of Cry3Bb1 resistance by western corn rootworm shows a pattern where resistance became prevalent across the agricultural landscape, likely evolving independently in multiple locations, and increased in magnitude over time (Gassmann et al. 2011, 2014; Shrestha et al. 2018; Gassmann 2021). A key question then becomes to what extent resistance to Gpp34/Tpp35Ab1 will follow a similar pattern. The evolution of resistance to Gpp34/Tpp35Ab1 across multiple states suggests that Gpp34/Tpp35Ab1 resistance by western corn rootworm has evolved independently in multiple locations (Gassmann et al. 2016, Ludwick et al. 2017, Reinders and Meinke 2022). It will be important to follow up this study by measuring inheritance and fitness costs in western corn rootworm strains with field-evolved resistance to Gpp34/Tpp35Ab1 corn to more accurately assess the risk of additional cases of resistance evolving in the field. Additionally, it will be important to use resistant and susceptible strains that are genetically similar.

Data on the dominance of resistance and fitness costs of resistance can be used to parameterize computer models aimed at building better strategies for insect resistance management to delay the evolution of resistance (Gould et al. 2006, Onstad and Meinke 2010, Haridas and Tenhumberg 2018). These models can aid in determining an appropriate refuge size and effects of current farming practices on resistance evolution. Once resistance becomes widespread in the landscape, it is difficult to manage, even in the absence of selection for resistance, because fitness costs typically take many generations to reduce the frequency of resistance alleles (St. Clair et al. 2020, Gassmann 2023). Furthermore, although fitness costs can aid in delaying resistance when resistance alleles are rare, once resistance becomes prevalent in a population, fitness costs have a minimal effect in delaying resistance, either in the case of single Bt traits or pyramids (Gould et al. 2006, Gassmann 2023). To preserve current and future transgenic technologies, it will be important to accurately assess the risk of resistance and implement appropriate strategies to mitigate the risk of resistance. For western corn rootworm, this may include increasing refuge size, diversifying management through approaches such as crop rotation and the use of non-Bt corn with soil-applied insecticides, and the use of transgenic crops with novel pyramids for which resistance alleles are rare for both traits in a pyramid (Roush 1998, Ellis et al. 2002, Moar et al. 2017, Wu et al. 2018, Carrière et al. 2020, Furlan et al. 2022, Gassmann 2023, Gassmann and Reisig 2023).

## References

[CIT0001] Abbott, W. S. 1925. A method of computing the effectiveness of an insecticide. J. Econ. Entomol. 18: 265–267.

[CIT0002] Andow, D. A., S. G. Pueppke, A. W. Schaafsma, A. J. Gassmann, T. W. Sappington, L. J. Meinke, P. D. Mitchell, T. M. Hurley, R. L. Hellmich, and R. P. Porter. 2016. Early detection and mitigation of resistance to *Bt* maize by western corn rootworm (Coleoptera: Chrysomelidae). J. Econ. Entomol. 109: 1–12. 26362989 10.1093/jee/tov238

[CIT0003] Bourguet, D., A. Genissel, and M. Raymond. 2000. Insecticide resistance and dominance levels. J. Econ. Entomol. 93: 1588–1595. 11142285 10.1603/0022-0493-93.6.1588

[CIT0004] Calles-Torrez, V., J. J. Knodel, M. A. Boetel, B. W. French, B. W. Fuller, and J. K. Ransom. 2019. Field-evolved resistance of northern and western corn rootworm (Coleoptera: Chrysomelidae) populations to corn hybrids expressing single and pyramided Cry3Bb1 and Cry34/35Ab1 Bt proteins in North Dakota. J. Econ. Entomol. 112: 1875–1886. 31114868 10.1093/jee/toz111

[CIT0005] Campagne, P., M. Kruger, R. Pasquet, B. le Ru, and J. van den Berg. 2013. Dominant inheritance of field-evolved resistance to Bt corn in *Busseola fusca*. PLoS One. 8: e69675. 23844262 10.1371/journal.pone.0069675 PMC3699669

[CIT0006] Carrière, Y., and B. Tabashnik. 2001. Reversing insect adaptation to transgenic insecticidal plants. Proc. R. Soc. B. 268: 1475–1480. 10.1098/rspb.2001.1689 PMC1088766 11454291

[CIT0007] Carrière, Y., C. Ellers-Kirk, K. Hartfield, G. Larocque, B. Degain, P. Dutilleul, T. J. Dennehy, S. E. Marsh, D. W. Crowder, X. Li, et al. 2012. Large-scale, spatially-explicit test of the refuge strategy for delaying insecticide resistance. Proc. Natl. Acad. Sci. U. S. A. 109: 775–780. 22215605 10.1073/pnas.1117851109 PMC3271916

[CIT0008] Carrière, Y., N. Crickmore, and B. E. Tabashnik. 2015. Optimizing pyramided transgenic Bt crops for sustainable pest management. Nat. Biotechnol. 33: 161–168. 25599179 10.1038/nbt.3099

[CIT0009] Carrière, Y., J. A. Fabrick, and B. E. Tabashnik. 2016. Can pyramids and seed mixtures delay resistance to Bt crops? Trends Biotechnol. 34: 291–302. 26774592 10.1016/j.tibtech.2015.12.011

[CIT0010] Carrière, Y., Z. Brown, S. Aglasan, P. Dutilleul, M. Carroll, G. Head, B. E. Tabashnik, P. S. Jørgensen, and S. P. Carroll. 2020. Crop rotation mitigates impacts of corn rootworm resistance to transgenic Bt corn. Proc. Natl. Acad. Sci. U. S. A. 117: 18385–18392. 32690686 10.1073/pnas.2003604117 PMC7414139

[CIT0011] Crickmore, N., C. Berry, S. Panneerselvam, R. Mishra, T. R. Connor, and B. C. Bonning. 2021. A structure-based nomenclature for *Bacillus thuringiensis* and other bacteria-derived pesticidal proteins. J. Invertebr. Pathol. 186: 107438. 32652083 10.1016/j.jip.2020.107438

[CIT0012] Deitloff, J., M. W. Dunbar, D. A. Ingber, B. E. Hibbard, and A. J. Gassmann. 2016. Effects of refuges on the evolution of resistance to transgenic corn by the western corn rootworm, *Diabrotica virgifera virgifera* LeConte. Pest Manage. Sci. 72: 190–198. 10.1002/ps.3988 25652190

[CIT0013] Dillen, K., P. D. Mitchell, T. van Looy, and E. Tollens. 2010. The western corn rootworm, a new threat to European agriculture: opportunities for biotechnology?. Pest Manage. Sci. 66: 95–966. 10.1002/ps.1966 20730987

[CIT0014] Ellis, R. T., B. A. Stockhoff, L. Stamp, H. E. Schnepf, G. E. Schwab, M. Knuth, J. Russell, G. A. Cardineau, and K. E. Narva. 2002. Novel *Bacillus thuringiensis* binary insecticidal crystal proteins active on western corn rootworm, *Diabrotica virgifera virgifera* LeConte. Sci. Rep. 68: 1137–1145. 10.1128/AEM.68.3.1137-1145.2002 PMC123759 11872461

[CIT0015] Furlan, L., F. Chiarini, B. Contiero, I. Benvegnù, F. G. Horgan, T. Kos, D. Lemić, and R. Bažok. 2022. Risk assessment and area-wide crop rotation to keep western corn rootworm below damage thresholds and avoid insecticide use in European maize production. Insects. 13: 415. 35621751 10.3390/insects13050415 PMC9145323

[CIT0016] García, M., C. García-Benítez, F. Ortego, and G. P. Farinós. 2023. Monitoring insect resistance to Bt maize in the European Union: update, challenges and future prospects. J. Econ. Entomol. doi: 10.1093/jee/toac154 PMC10125040 36610405

[CIT0017] Gassmann, A. J. 2016. Resistance to Bt maize by western corn rootworm: insights from the laboratory and the field. Curr. Opin. Insect Sci. 15: 111–115. 27436740 10.1016/j.cois.2016.04.001

[CIT0018] Gassmann, A. J. 2021. Resistance to Bt maize by western corn rootworm: effects of pest biology, the pest–crop interaction and the agricultural landscape on resistance. Insects. 12: 136. 33562469 10.3390/insects12020136 PMC7915852

[CIT0019] Gassmann, A. J. 2023. Fitness costs of resistance and their potential application for insect resistance management, pp. 465–492. *In* D. W. Onstad and L. M. Knolhoff (eds.), Insect resistance management: biology, economics and predictions, 3rd ed. Elsevier, London.

[CIT0020] Gassmann, A. J., Y. Carrière, and B. E. Tabashnik. 2009. Fitness costs of insect resistance to *Bacillus thuringiensis*. Annu. Rev. Entomol. 54: 147–163. 19067630 10.1146/annurev.ento.54.110807.090518

[CIT0021] Gassmann, A. J., and D. D. Reisig. 2023. Management of insect pests with Bt crops in the United States. Annu. Rev. Entomol. 68: 31–49. 36170641 10.1146/annurev-ento-120220-105502

[CIT0022] Gassmann, A. J., J. L. Petzold-Maxwell, R. S. Keweshan, and M. W. Dunbar. 2011. Field-evolved resistance to Bt maize by western corn rootworm. PLoS One. 6: e22629. 21829470 10.1371/journal.pone.0022629 PMC3146474

[CIT0023] Gassmann, A. J., J. L. Petzold-Maxwell, E. H. Clifton, M. W. Dunbar, A. M. Hoffmann, D. A. Ingber, and R. S. Keweshan. 2014. Field-evolved resistance by western corn rootworm to multiple *Bacillus thuringiensis* toxins in transgenic maize. Proc. Natl. Acad. Sci. U. S. A. 111: 5141–5146. 24639498 10.1073/pnas.1317179111 PMC3986160

[CIT0024] Gassmann, A. J., R. B. Shrestha, S. R. K. Jakka, M. W. Dunbar, E. H. Clifton, A. R. Paolino, D. A. Ingber, B. W. French, K. E. Masloski, J. W. Dounda, et al. 2016. Evidence of resistance to Cry34/35Ab1 corn by western corn rootworm (Coleoptera: Chrysomelidae): root injury in the field and larval survival in plant-based bioassays. J. Econ. Entomol. 109: 1872–1880. 27329619 10.1093/jee/tow110

[CIT0025] Gassmann, A. J., R. B. Shrestha, A. L. Kropf, C. R. St Clair, and B. D. Brenizer. 2020. Field‐evolved resistance by western corn rootworm to Cry34/35Ab1 and other *Bacillus thuringiensis* traits in transgenic maize. Pest Manage. Sci. 76: 268–276. 10.1002/ps.5510 31207042

[CIT0026] Gould, F. 1998. Sustainability of transgenic insecticidal cultivars: integrating pest genetics and ecology. Annu. Rev. Entomol. 43: 701–726. 15012402 10.1146/annurev.ento.43.1.701

[CIT0027] Gould, F., M. B. Cohen, J. S. Bentur, G. G. Kennedy, and J. VanDuyn. 2006. Impact of small fitness costs on pest adaptation to crop varieties with multiple toxins: a heuristic model. J. Econ. Entomol. 99: 2091–2099. 17195678 10.1603/0022-0493-99.6.2091

[CIT0028] Gray, M. E., and K. L. Steffey. 1998. Corn rootworm (Coleoptera: Chrysomelidae) larval injury and root compensation of 12 maize hybrids: an assessment of the economic injury index. J. Econ. Entomol. 91: 723–740.

[CIT0029] Gray, M. E., T. W. Sappington, N. J. Miller, J. Moeser, and M. O. Bohn. 2009. Adaptation and invasiveness of western corn rootworm: intensifying research on a worsening pest. Annu. Rev. Entomol. 54: 303–321. 19067634 10.1146/annurev.ento.54.110807.090434

[CIT0030] Groeters, F. R., and B. E. Tabashnik. 2000. Roles of selection intensity, major genes, and minor genes in evolution of insecticide resistance. J. Econ. Entomol. 93: 1580–1587. 11142284 10.1603/0022-0493-93.6.1580

[CIT0031] Hammack, L., and B. W. French. 2007. Sexual dimorphism of basitarsi in pest species of Diabrotica and Cerotoma (Coleoptera: Chrysomelidae). Ann. Entomol. Soc. Am. 100: 59–63.

[CIT0032] Haridas, C. V., and B. Tenhumberg. 2018. Modeling effects of ecological factors on evolution of polygenic pesticide resistance. J. Theor. Biol. 456: 224–232. 30075171 10.1016/j.jtbi.2018.07.034

[CIT0033] Hoffmann, A. M., B. W. French, S. T. Jaronski, and A. J. Gassmann. 2014. Effects of entomopathogens on mortality of western corn rootworm and fitness costs of resistance to Cry3Bb1 maize. J. Econ. Entomol. 107: 352–360. 24665720 10.1603/ec13247

[CIT0034] Hoffmann, A. M., B. W. French, R. L. Hellmich, N. Lauter, and A. J. Gassmann. 2015. Fitness costs of resistance to Cry3Bb1 maize by western corn rootworm. J. Appl. Entomol. 139: 403–415.

[CIT0035] Huang, F. 2021. Resistance of the fall armyworm, *Spodoptera frugiperda*, to transgenic *Bacillus thuringiensis* Cry1F corn in the Americas: lessons and implications for Bt corn IRM in China. Insect Sci. 28: 574–589. 32478944 10.1111/1744-7917.12826

[CIT0036] Huang, F., D. A. Andow, and L. L. Buschman. 2011. Success of the high-dose/refuge resistance management strategy after 15 years of Bt crop use in North America. Entomol. Exp. Appl. 140: 1–16.

[CIT0037] Ingber, D. A., and A. J. Gassmann. 2015. Inheritance and fitness costs of resistance to Cry3Bb1 corn by western corn rootworm (Coleoptera: Chrysomelidae). J. Econ. Entomol. 108: 2421–2432. 26453731 10.1093/jee/tov199

[CIT0038] Jakka, S. R. K., R. B. Shrestha, and A. J. Gassmann. 2016. Broad-spectrum resistance to *Bacillus thuringiensis* toxins by western corn rootworm (*Diabrotica virgifera virgifera*). Sci. Rep. 6: 27860. 27297953 10.1038/srep27860 PMC4906537

[CIT0039] Kelker, M. S., C. Berry, S. L. Evans, R. Pai, D. G. McCaskill, N. X. Wang, J. C. Russell, M. D. Baker, C. Yang, J. W. Pflugrath, et al. 2014. Structural and biophysical characterization of *Bacillus thuringiensis* insecticidal proteins Cry34Ab1 and Cry35Ab1. PLoS One. 9: e112555. 25390338 10.1371/journal.pone.0112555 PMC4229197

[CIT0040] Levine, E., J. L. Spencer, S. A. Isard, D. W. Onstad, and M. E. Gray. 2002. Adaptation of the western corn rootworm to crop rotation: evolution of a new strain in response to a management practice. Am. Entomol. 48: 94–107.

[CIT0041] Liu, Y., and B. E. Tabashnik. 1997. Inheritance of resistance to the *Bacillus thuringiensis* toxin Cry1C in the diamondback moth. Appl. Environ. Microbiol. 63: 2218–2223. 16535623 10.1128/aem.63.6.2218-2223.1997 PMC1389178

[CIT0042] Ludwick, D. C., L. N. Meihls, K. R. Ostlie, B. D. Potter, L. French, and B. E. Hibbard. 2017. Minnesota field population of western corn rootworm (Coleoptera: Chrysomelidae) shows incomplete resistance to Cry34Ab1/Cry35Ab1 and Cry3Bb1. J. Appl. Entomol. 141: 28–40.

[CIT0043] Matten, S. R., R. J. Frederick and A. H. Reynolds. 2012. United States Environmental Protection Agency insect resistance management programs for plant-incorporated protectants and use of simulation modeling, pp. 175–267. *In* C. Wozniak and A. McHughen (eds.), Regulation of agricultural biotechnology: the United States and Canada. Springer Netherlands, Dordrecht.

[CIT0044] Meihls, L. N., M. L. Higdon, B. D. Siegfried, N. J. Miller, T. W. Sappington, M. R. Ellersieck, T. A. Spencer, and B. E. Hibbard. 2008. Increased survival of western corn rootworm on transgenic corn within three generations of on-plant greenhouse selection. Proc. Natl. Acad. Sci. U. S. A. 105: 19177–19182. 19047626 10.1073/pnas.0805565105 PMC2614735

[CIT0045] Meinke, L. J., B. D. Siegfried, R. J. Wright, and L. D. Chandler. 1998. Adult susceptibility of Nebraska western corn rootworm (Coleoptera: Chrysomelidae) populations to selected insecticides. J. Econ. Entomol. 91: 594–600.

[CIT0046] Meinke, L. J., T. W. Sappington, D. W. Onstad, T. Guillemaud, N. J. Miller, J. Komáromi, N. Levay, L. Furlan, J. Kiss, and F. Toth. 2009. Western corn rootworm (*Diabrotica virgifera virgifera* LeConte) population dynamics. Agric. For. Entomol. 11: 29–46.

[CIT0047] Meinke, L. J., D. Souza, and B. D. Siegfried. 2021. The use of insecticides to manage the western corn rootworm, *Diabrotica virgifera virgifera*, LeConte: history, field-evolved resistance, and associated mechanisms. Insects. 12: 112. 33525337 10.3390/insects12020112 PMC7911631

[CIT0048] Moar, W., C. Khajuria, M. Pleau, O. Ilagan, M. Chen, C. Jiang, P. Price, B. McNulty, T. Clark, and G. Head. 2017. Cry3Bb1-resistant western corn rootworm, *Diabrotica virgifera virgifera* (LeConte) does not exhibit cross-resistance to DvSnf7 dsRNA. PLoS One. 12: e0169175. 28060922 10.1371/journal.pone.0169175 PMC5217956

[CIT0049] Murphy, A. F., and C. H. Krupke. 2011. Mating success and spermatophore composition in western corn rootworm (Coleoptera: Chrysomelidae). Environ. Entomol. 40: 1585–1594. 22217777 10.1603/EN11137

[CIT0050] Oleson, J. D., Y. -L. Park, T. M. Nowatzki, and J. J. Tollefson. 2005. Node-injury scale to evaluate root injury by corn rootworms (Coleoptera: Chrysomelidae). J. Econ. Entomol. 98: 1–8. 15765660 10.1093/jee/98.1.1

[CIT0051] Onstad, D. W., and L. J. Meinke. 2010. Modeling evolution of *Diabrotica virgifera virgifera* (Coleoptera: Chrysomelidae) to transgenic corn with two insecticidal traits. J. Econ. Entomol. 103: 849–860. 20568632 10.1603/ec09199

[CIT0052] Paddock, K. J., B. E. Hibbard, J. Barry, A. Sethi, A. L. Mueller, K. S. Shelby, and A. E. Pereira. 2021. Restoration of susceptibility following removal of selection for Cry34/35Ab1 resistance documents fitness costs in resistant population of western corn rootworm, *Diabrotica virgifera virgifera*. Pest Manage. Sci. 77: 2385–2394. 10.1002/ps.6266 33415809

[CIT0053] Paolino, A. R., and A. J. Gassmann. 2017. Assessment of inheritance and fitness costs associated with field-evolved resistance to Cry3Bb1 maize by western corn rootworm. Toxins. 9: 159. 28492498 10.3390/toxins9050159 PMC5450707

[CIT0054] Petzold-Maxwell, J. L., X. Cibils-Stewart, B. W. French, and A. J. Gassmann. 2012. Adaptation by western corn rootworm (Coleoptera: Chrysomelidae) to Bt maize: inheritance, fitness costs, and feeding preference. J. Econ. Entomol. 105: 1407–1418. 22928323 10.1603/ec11425

[CIT0055] Petzold-Maxwell, J. L., S. T. Jaronski, E. H. Clifton, M. W. Dunbar, M. A. Jackson, and A. J. Gassmann. 2013. Interactions among Bt maize, entomopathogens and rootworm species (Coleoptera: Chrysomelidae) in the field: effects on survival, yield and root injury. J. Econ. Entomol. 106: 622–632. 23786047 10.1603/ec12375

[CIT0056] Price, P. W., C. E. Bouton, P. Gross, B. A. McPheron, J. N. Thompson, and A. E. Wesi. 1980. Interactions among three trophic levels: influence of plants on interactions between insect herbivores and natural enemies. Ann. Rev. Ecol. Syst. 11: 41–65.

[CIT0057] Reinders, J. D., and L. J. Meinke. 2022. Reduced susceptibility of western corn rootworm (*Diabrotica virgifera virgifera* LeConte) populations to Cry34/35Ab1-expressing maize in northeast Nebraska. Sci. Rep. 12: 19221. 36357469 10.1038/s41598-022-23755-z PMC9649616

[CIT0058] Reinders, J. D., E. E. Reinders, E. A. Robinson, B. W. French, and L. J. Meinke. 2022. Evidence of western corn rootworm (*Diabrotica virgifera virgifera* LeConte) field‐evolved resistance to Cry3Bb1 + Cry34/35Ab1 maize in Nebraska. Pest Manage. Sci. 78: 1356–1366. 10.1002/ps.6752 34873825

[CIT0059] Roush, R. T. 1997. Bt-transgenic crops: just another pretty insecticide or a chance for a new start in resistance management? Pestic. Sci. 51: 328–334.

[CIT0060] Roush, R. T. 1998. Two–toxin strategies for management of insecticidal transgenic crops: can pyramiding succeed where pesticide mixtures have not?. Philos. Trans. R. Soc. B. 353: 1777–17866.

[CIT0061] Sanahuja, G., R. Banakar, R. M. Twyman, T. Capell, and P. Christou. 2011. *Bacillus thuringiensis*: a century of research, development and commercial applications. Plant Biotechnol. J. 9: 283–300. 21375687 10.1111/j.1467-7652.2011.00595.x

[CIT0062] Santiago‐González, J. C., D. L. Kerns, G. P. Head, and F. Yang. 2022. Effective dominance and redundant killing of single‐ and dual‐gene resistant populations of *Helicoverpa zea* on pyramided Bt corn and cotton. Pest Manage. Sci. 78: 4333–4339. 10.1002/ps.7052 35750998

[CIT0063] SAS Institute. 2015. SA Software V9.4. Cary, NC: SAS Institute Inc.

[CIT0064] Schneider, C. A., W. S. Rasband, and K. W. Eliceiri. 2012. NIH image to ImageJ: 25 years of image analysis. Nat. Methods. 9: 671–675. 22930834 10.1038/nmeth.2089 PMC5554542

[CIT0065] Shrestha, R. B., and A. J. Gassmann. 2020. Inheritance and fitness costs of Cry3Bb1 resistance in diapausing field strains of western corn rootworm (Coleoptera: Chrysomelidae). J. Econ. Entomol. 113: 2873–2882. 32990316 10.1093/jee/toaa213 PMC7724752

[CIT0066] Shrestha, R. B., S. R. K. Jakka, B. W. French, and A. J. Gassmann. 2016. Field-based assessment of resistance to Bt corn by western corn rootworm (Coleoptera: Chrysomelidae). J. Econ. Entomol. 109: 1399–1409. 27122498 10.1093/jee/tow087

[CIT0067] Shrestha, R. B., M. W. Dunbar, B. W. French, and A. J. Gassmann. 2018. Effects of field history on resistance to Bt maize by western corn rootworm, *Diabrotica virgifera virgifera* LeConte (Coleoptera: Chrysomelidae). PLoS One. 13: e0200156. 29969492 10.1371/journal.pone.0200156 PMC6029802

[CIT0068] Siegfried, B. D., and R. L. Hellmich. 2012. Understanding successful resistance management. GM Crops Food. 3: 184–193. 22688691 10.4161/gmcr.20715

[CIT0069] Smith, J. L., Y. Farhan, and A. W. Schaafsma. 2019. Practical resistance of *Ostrinia nubilalis* (Lepidoptera: Crambidae) to Cry1F *Bacillus thuringiensis* maize discovered in Nova Scotia, Canada. Sci. Rep. 9: 18247. 31796764 10.1038/s41598-019-54263-2 PMC6890797

[CIT0070] Sokal, R. R., and F. J. Rohlf. 1995. Biometry, 3rd ed. W. H. Freeman and Company, New York

[CIT0071] St. Clair, C. R., E. H. Clifton, M. W. Dunbar, K. E. Masloski, A. R. Paolino, R. B. Shrestha, and A. J. Gassmann. 2020. Applying a selection experiment to test for fitness costs of Bt resistance in western corn rootworm (Coleoptera: Chrysomelidae) and the effect of density on fitness costs. J. Econ. Entomol. 113: 2473–2479. 32772116 10.1093/jee/toaa168 PMC7717071

[CIT0072] Storer, N. P., J. M. Babcock, and J. M. Edwards. 2006. Field measures of western corn rootworm (Coleoptera: Chrysomelidae) mortality caused by Cry34/35Ab1 proteins expressed in maize event 59122 and implications for trait durability. J. Econ. Entomol. 99: 1381–1387. 16937696 10.1603/0022-0493-99.4.1381

[CIT0073] Tabashnik, B. E. 1994. Delaying insect adaptation to transgenic plants: seed mixtures and refugia reconsidered. Proc. R. Soc. B. 255: 7–12.

[CIT0074] Tabashnik, B. E., and Y. Carrière. 2014. Successes and failures of transgenic Bt crops: global patterns of field-evolved resistance. *In* M. Soberón, A. Gao, and A. Bravo (eds.), Bt resistance: characterization and strategies for GM crops producing *Bacillus Thuringiensis* toxins. CABI, Wallingford.

[CIT0075] Tabashnik, B. E., and F. Gould. 2012. Delaying corn rootworm resistance to Bt corn. J. Econ. Entomol. 105: 767–776. 22812111 10.1603/ec12080

[CIT0076] Tabashnik, B. E., F. Gould, and Y. Carrière. 2004. Delaying evolution of insect resistance to transgenic crops by decreasing dominance and heritability. J. Evol. Biol. 17: 904–912. 15271091 10.1111/j.1420-9101.2004.00695.x

[CIT0077] Tabashnik, B. E., A. J. Gassmann, D. W. Crowder, and Y. Carriére. 2008. Insect resistance to Bt crops: evidence versus theory. Nat. Biotechnol. 26: 199–202. 18259177 10.1038/nbt1382

[CIT0078] Tabashnik, B. E., J. B. J. van Rensburg, and Y. Carrière. 2009. Field-evolved insect resistance to Bt crops: definition, theory, and data. J. Econ. Entomol. 102: 2011–2025. 20069826 10.1603/029.102.0601

[CIT0079] Tabashnik, B. E., T. Brévault, and Y. Carrière. 2013. Insect resistance to Bt crops: lessons from the first billion acres. Nat. Biotechnol. 31: 510–521. 23752438 10.1038/nbt.2597

[CIT0080] Tinsley, N. A., J. L. Spencer, R. E. Estes, J. R. Prasifka, P. M. Schrader, B. W. French, and M. E. Gray. 2015. Larval mortality and development for rotation-resistant and rotation-susceptible populations of western corn rootworm on Bt corn. J. Appl. Entomol. 139: 46–54.

[CIT0081] Toepfer, S., and U. Kuhlmann. 2006. Constructing life-tables for the invasive maize pest *Diabrotica virgifera virgifera* (Col.; Chrysomelidae) in Europe. J. Appl. Entomol. 130: 193–205.

[CIT0082] US EPA. 1998. Final report of the FIFRA scientific advisory panel subpanel on *Bacillus thuringiensis* (Bt) plant-pesticides and resistance management (https://archive.epa.gov/scipoly/sap/meetings/web/pdf/finalfeb.pdf) (Accessed 28 October 2022).

[CIT0083] US EPA. 2010a. Biopesticides registration action document event mon863 *Bacillus thuringiensis* Cry3bb1 corn (https://www3.epa.gov/pesticides/chem_search/reg_actions/pip/cry3bb1-brad.pdf) (Accessed 10 December 2022).

[CIT0084] US EPA. 2010b. Biopesticides registration action document modified Cry3A protein and the genetic material necessary for its production (Via Elements of pZM26) in event MIR604 corn SYN-IR604-8 (https://www3.epa.gov/pesticides/chem_search/reg_actions/pip/mcry3a-brad.pdf) (Accessed 10 December 2022).

[CIT0085] US EPA. 2010c. Biopesticides registration action document *Bacillus thuringiensis* Cry34Ab1 and Cry35Ab1 proteins and the genetic material necessary for their production (PHP17662 T-DNA) in event DAS-59122-7 corn (OECD unique identifier: DAS-59122-7) (https://www3.epa.gov/pesticides/chem_search/reg_actions/pip/cry3435ab1-brad.pdf) (Accessed 10 December 2022).

[CIT0086] US EPA. 2012. Draft biopesticides registration action document *Bacillus thuringiensis* eCry3.1Ab insecticidal protein and genetic material (https://www.regulations.gov/document/EPA-HQ-OPP-2012-0108-0010) (Accessed 10 December 2022).

[CIT0087] US EPA. 2021. Insect resistance management for Bt plant-incorporated protectants. (https://www.epa.gov/regulation-biotechnology-under-tsca-and-fifra/insect-resistance-management-bt-plant-incorporated) (Accessed 28 October 2022).

[CIT0088] Vidal, S., U. Kuhlmann, and C. R. Edwards. 2005. Western corn rootworm: ecology and management. CABI, Wallingford.

[CIT0089] Wangila, D. S., A. J. Gassmann, J. L. Petzold-Maxwell, B. W. French, and L. J. Meinke. 2015. Susceptibility of Nebraska western corn rootworm (Coleoptera: Chrysomelidae) populations to Bt corn events. J. Econ. Entomol. 108: 742–751. 26470186 10.1093/jee/tou063

[CIT0090] Wechsler, S., and D. Smith. 2018. Has resistance taken root in U.S. corn fields? Demand for insect control. J. Agric. Econ. 100: 1136–1150.

[CIT0091] Wu, K., C. E. Taylor, E. Fishilevich, K. E. Narva, and B. D. Siegfried. 2018. Rapid and persistent RNAi response in western corn rootworm adults. Pestic. Biochem. Physiol. 150: 66–70. 30195389 10.1016/j.pestbp.2018.07.002

[CIT0092] Yang, F., G. P. Head, P. A. Price, J. C. Santiago González, and D. L. Kerns. 2020. Inheritance of *Bacillus thuringiensis* Cry2Ab2 protein resistance in *Helicoverpa zea* (Lepidoptera: Noctuidae). Pest Manage. Sci. 76: 3676–3684. 10.1002/ps.5916 32419321

[CIT0093] Zukoff, S. N., K. R. Ostlie, B. Potter, L. N. Meihls, A. L. Zukoff, L. French, M. R. Ellersieck, B. Wade French, and B. E. Hibbard. 2016. Multiple assays indicate varying levels of cross resistance in Cry3Bb1-selected field populations of the western corn rootworm to mCry3A, eCry3.1Ab, and Cry34/35Ab1. J. Econ. Entomol. 109: 1387–1398. 27106225 10.1093/jee/tow073

